# The Effect of Xenon on the Voltage‒Current Characteristics of Tethered Lipid Bilayers

**DOI:** 10.1007/s00232-025-00346-3

**Published:** 2025-04-28

**Authors:** Hadeel Alobeedallah, Bruce Cornell, Hans Coster

**Affiliations:** 1 School of Chemical and Biomolecular Engineering, The University of Sydney, Sydney, 2006 Australia; 2 SDx Tethered Membranes Pty Ltd, Roseville, NSW 2069 Australia

**Keywords:** Tethered lipid membranes, Voltage‒current (V‒I) characteristics, Activation energy

## Abstract

In this study, we describe the effect of the noble gas, xenon on the electrical properties of tethered lipid bilayer membranes, (tBLMs), including the effect of xenon on the activation energy for electrical conduction through the tBLM. Such studies benefit from the stability of a tethered membrane given the wide range of temperatures that are scanned and the time required for these measurements. The results indicate that xenon increases the activation energy for electrical conduction through bilayers and decreases the average pore size that dominates the electrical conductance of the lipid bilayers at low voltages. Xenon possesses a high affinity for lipid membranes and is a potent general anaesthetic. Its anaesthetic potency is possibly associated with its effects on proteins embedded in the lipid membranes.

**Graphical Abstract:**

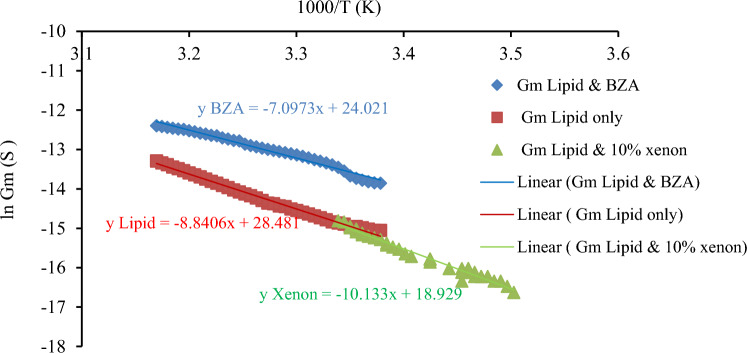

## Introduction

General anaesthetics are often defined clinically as compounds that induce a reversible loss of consciousness, awareness and sensation required during surgical procedures. General anaesthetics include various compounds that are structurally diverse, such as halothanes, ether, xenon, barbiturates, alphaxalone and chloral hydrate. Volatile anaesthetic gases have been widely used in clinical applications and are considered among the most important medical advances. On the other hand, they are also considered the most dangerous drugs that physicians currently use. The importance of general anaesthetic gases arises from the procedures involved in their administration and the mechanisms by which they act. Hence, understanding their mechanisms of action at the fundamental, molecular level is useful.

Many studies have investigated the mechanisms underlying the action of general anaesthetics; however, the physical mechanisms by which anaesthetic agents induce an anaesthetic state remain poorly understood. Some possible mechanisms of action have been reviewed by Eckenhoff (2001). They reviewed various possible mechanisms of action for anesthetic agents and focused on how these agents exert their effects at the molecular level. Some of the primary mechanisms discussed by Eckenhoff et al. includes ion Channel modulation and membrane lipid Interactions. A study by Zizzi et al. (2022) employed molecular docking simulations to explore the binding interactions between human tubulin and four volatile anesthetics: ethylene, desflurane, halothane, and methoxyflurane. The research identified specific binding sites on various tubulin isotypes and ranked the binding affinities of these anesthetics. The findings suggest that these anesthetics can transiently bind to tubulin, potentially influencing microtubule dynamics and neuronal function. Further investigations by Zizzi et al. (2021) examined the effects of volatile anesthetics on both phospholipid membranes and intracellular tubulin. Through computational molecular modeling, the study demonstrated that anesthetics rapidly partition into the membrane and significantly alter its mechanical properties.

The present study focuses on the membrane hypothesis (membrane bilayer-mediated mechanism), which states that general anaesthetics act by targeting the structural and dynamic properties of lipid membranes. These changes affect the structure and function of proteins embedded in lipid membranes.

Xenon is an odorless, colorless and chemically unreactive gas. It is the rarest noble gas and is present in atmospheric air at a concentration of no more than 0.086 ppm. Despite the inert properties of xenon, it has the ability to interact with cellular proteins and cell membrane elements. This interaction is encouraged by an induced dipole that is created primarily by the polarisation of the large unique electron shell of xenon by nearby molecules (Lynch et al. 2000). The ability of xenon to interact with cellular proteins and cell membrane elements is the key factor underlying its anaesthetic potency.

Most of the studies that have investigated the effect of xenon on lipid bilayers agree that xenon is preferentially localised in the hydrophobic core of the bilayer. This is due to the hydrophobic properties of xenon. Chen et al. (2015) conducted molecular dynamic simulations to compare the effects of four kinds of noble gases (Ne, Ar, Kr and Xe) on phospholipid bilayers and reported that xenon molecules had the strongest effect on these membranes, with the strongest preference for localising in the hydrophobic core of the bilayer. In addition, Booker and Sum (2013) investigated the interactions of xenon with model phospholipid membranes via molecular dynamic simulations. These results agreed with those of Chen et al. (2015), who also reported that xenon localised in the hydrophobic core of the bilayer. In addition, they reported that increasing the concentration of xenon caused a corresponding increase in the ordering of the lipid tails, resulting in a relative decrease in membrane fluidity. A study by Rózsa et al. (2023) however, suggested that Xenon may not be located in the lipid bilayer interior but closer to the bilayer head groups and that this alters the lipid packing and leads to swelling of the bilayer. They also reported that the order of the acyl chains within the bilayer was slightly enhanced, suggesting a more ordered structure in the presence of xenon.

Membrane swelling was also observed upon the incorporation of xenon (Booker and Sum 2013). This was again due to the localisation of xenon molecules in the core of the lipid bilayer, which caused a substantial increase in the membrane thickness and an increase in the gaps between the terminal carbons of the hydrocarbon acyl chains.

In another interesting study conducted by Yamamoto et al. (2012), the diffusive nature of xenon in the structure of the lipid membrane was investigated. These authors performed molecular dynamic simulations of a 1-palmitoyl-2-oleoyl phosphatidylethanolamine bilayer with and without Xenon molecules at several pressures (Yamamoto et al. 2012). That study emphasized the importance of specific neurotransmitter systems, brain wave activity, and synaptic plasticity in the mechanism of anaesthetic action. Weinrich and Worcester (2013) also studied the effects of Xenon molecules on lipid membranes via lamellar neutrons and X-ray diffraction which demonstrated that Xenon changed the phase distribution of the lipid raft mixtures to favour the disordered phase. On the other hand, Petrov et al. (2018) showed that Xenon in black lipid bilayers affects mechanosensitive ion channels and attributed that to Xenon-protein interactions.

A number of studies that have investigated the effects of xenon on lipid bilayers have employed molecular dynamic simulations. Although this method is mature, it has not yet realised the full potential of the best algorithms, long-range interactions and transferable polarizable force fields; there are many gaps to be filled (Berendsen 1999). Moreover, Ingólfsson et al. (2011) reported that molecular dynamic simulation is less successful in quantitatively predicting single-channel conductance. In addition, previous studies on the effects of xenon on lipid bilayers involved planar free-standing BLM as a membrane model system. As discussed previously, these membrane models are very fragile and limit the characterisation techniques that can be used.

In this study, a new experimental platform using tethered lipid bilayers (tBLM) was designed and employed to investigate the effect of xenon on lipid membranes.

## Experimental Procedures

### Materials

**Fig. 1:**
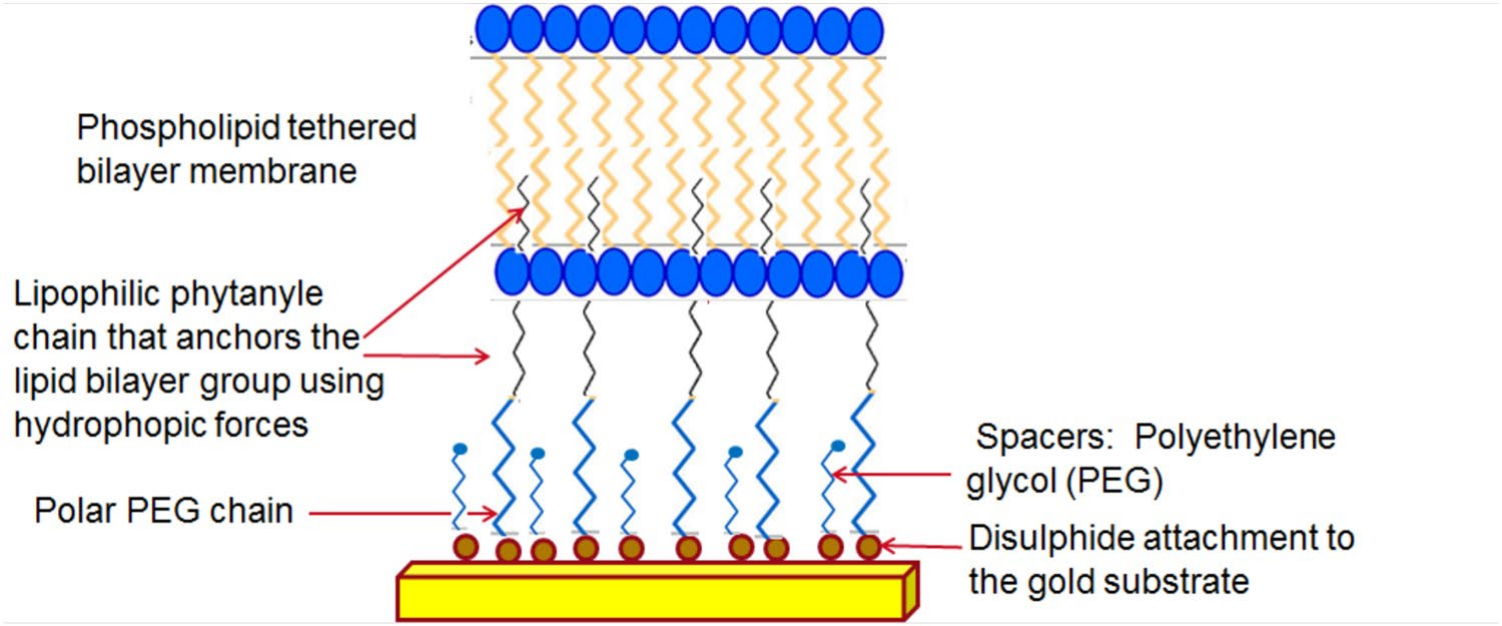
Basic structure of tethered lipid bilayers

The present study utilises the tethered lipid bilayer Am199 (a mixture of diphytanyl ether phosphatidylcholine and glycerodiphytanylether at a molar ratio of 70:30). Xenon, Am215 (gramicidin A + lipid), ethanol (99.9%) and isotonic phosphate-buffered saline (PBS) were obtained from Sigma Aldrich. All reagents were of the highest grade commercially available. The chips on which the tethered membranes were generated were manufactured by SDx® Tethered Membranes Pty Ltd. The SDx® chip comprises a six-electrode polycarbonate slide that contains a 100 nm thick reference gold electrode in contrast to six separate isolated gold film electrodes of 0.7 × 3 mm each in a 100 µm high-flow cell (Alobeedallah et al. 2016). The tethered lipid bilayers are formed on these gold electrodes. A schematic of the basic structure of the tethered lipid bilayers on the gold electrode is presented in Fig. 1.

## Methods

### Xenon/Tethered Lipid Membrane Preparation

The tethered membranes were constructed on an SDx® chip (Hoiles et al. 2014).

The first stage involves coating the freshly deposited gold electrode in the SDX chip with a 1 mM monolayer layer of benzyl disulfide diphytanyl bisethylene glycol tethers and benzyl disulfide tetraethylene glycol spacers. Varying the ratio of tethers: spacers in the coating mix layer can greatly affect the structure of the bilayer by controlling the available space for phospholipid lateral movement. In this study, three different densities were investigated: T1 (1% tethers), T10 (10% tethers) and T100 (100% tethers). However, a tethering density of 10% (T10) was used for the majority of the tethered membrane experiments conducted in this work, as it provides both hydration and conformational freedom for the bilayer lipids.

To prepare the xenon/PBS solutions and achieve the optimal levels of xenon saturation in the PBS solution, a new apparatus containing two main components was constructed. First, a syringe-valve vacuum system was connected to a vial at one end and to the injection dosing system at the other end. This component was responsible for evacuating the air from the vial so that the concentration of xenon was relatively high. The second component of the apparatus was the injection dosing system, which consisted of another syringe-valve system connected to a xenon source inlet at one end and to the vial at the other end. This component provided an automatic mechanism to regulate the volume of xenon injected and was controlled by the pressure inside the vial. The injection system involves an automatic dosing syringe that injects a known volume of xenon into the vial where it dissolves in PBS. This process was conducted at 10 °C to increase the solubility of xenon in PBS. When all the xenon volume was injected into the vial, the vial was carefully disconnected and left at room temperature to warm the xenon/PBS mixture before injecting it into the assembled tethered lipid membranes. This step was crucial to avoid any possible changes in the lipid temperature that might occur due to the addition of the xenon/PBS mixture. The thickness of the bilayer, the area per lipid value, or the order parameter are all governed by temperature (Nagle and Tristram 2000; Garcia et al. 2005). After the xenon/PBS mixture reached room temperature, 10 µl of xenon/PBS solution was injected into the assembled tBLM in the SDx® chip. Xenon/PBS solutions of 10 and 30% saturation were prepared and injected into the tBLM. The amount of xenon required to be dissolved in the PBS to prepare the required concentrations was calculated from the gas laws.

### Electrical Measurements

Voltage–current (V–I) measurements were performed via a recent approach developed by Cranfield et al. (2014). Individual ramped triangular potentials from zero to 500 mV with a period of 2–10 ms were applied. Voltage increases occurred stepwise at 5 mV/0.05 ms. For each 0.05 ms step, current recordings were averaged over 0.01 ms intervals after the voltage increment. The current pulses are short in duration, and while the gold electrodes to which the bilayers are tethered are “blocking” electrodes for DC currents, the electrode capacitance is such that for short current pulses, the electrode impedance is relatively small. The method was explained in detail in our previous work (Alobeedallah et al. 2020, 2022a, b).

### Conductance–Temperature Dependence Measurements

In the present study, the activation energy for electrical conduction through lipid bilayers was determined from Arrhenius conductivity plots of the conductivity as a function of temperature. The method was explained in detail in our previous work (Alobeedallah et al. 2022a, b). The Born energy, average pore radius and pore conductivity of lipid membranes with BZA incorporated were also calculated (see Appendix A).

The temperature control apparatus that was used in the previous experiments with BZA and cholesterol (Alobeedallah et al. 2020, 2022a, b, 2023) could not be used to obtain conductance–temperature dependence curves of tethered membranes with xenon, as the heating process used in this apparatus was not suitable for studies conducted on tBLM with xenon due to the low solubility of xenon at high temperatures. Xenon tends to show better solubility in lipid membranes at lower temperatures (Yeh and Peterson 1963). The temperature control system that was used to obtain the conductance–temperature dependency data involved a cooling protocol instead of heating. This was implemented in the laboratory using a refrigerator fan, an SDx® TethaPod unit and a digital thermometer. The cooling process was conducted from room temperature (22 °C) to almost 10 °C, and a refrigerator fan system was used to achieve this. The TethaPod unit containing the SDx® electrode with the formed membranes was placed inside an insulated box placed in front of the fan; the cold air generated by the fan cooled the membranes, gradually incorporating xenon. A thermometer was placed inside the insulated box to monitor the temperature of the membrane. The membrane conductance was measured for every degree decrease in the membrane temperature.

## Results and Discussion

### Voltage–Current Characteristic Measurements

**Fig. 2:**
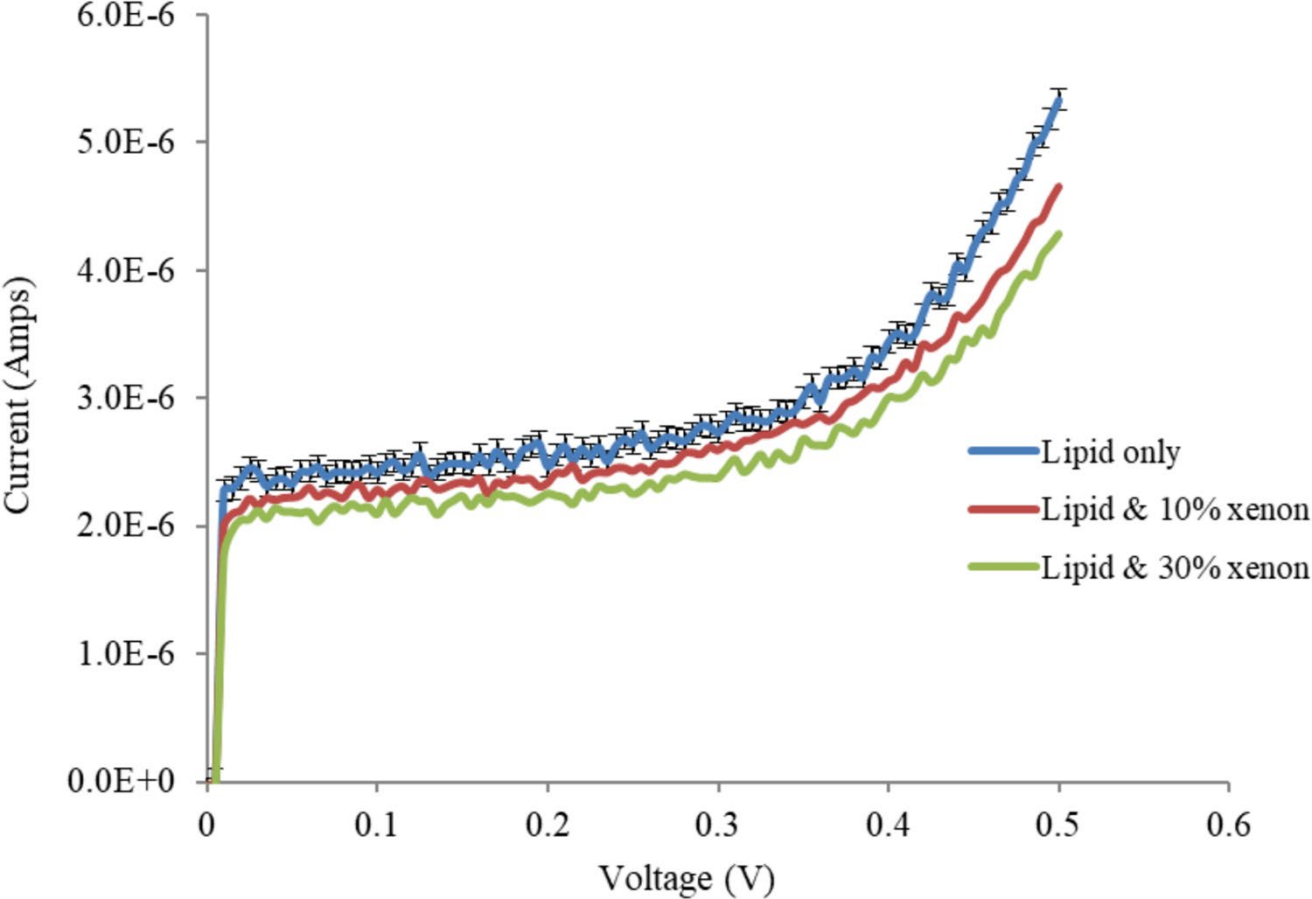
Effects of xenon on the V‒I curves of T10 (10% tethers) Am199 bilayers. Error bars represent the standard deviation calculated from four experimental runs. Error bars are shown for one data set to enhance figure clarity

Experiments were conducted to study the effects of different concentrations of xenon on the V‒I characteristics and electrical properties of tethered membranes on the basis of the methods described in our previous work (Alobeedallah et al. 2022a, b). Figure 2 shows that the incorporation of xenon into the tethered lipid membrane caused a decrease in membrane conductance.

Figure 2 also shows that the initial current step, which is due to the capacitive charging current, is decreased by xenon, suggesting that the membrane capacitance has decreased. The membrane capacitance was calculated via the V‒I curve shown in Fig. 2. Given that *Q* = *Cm* × *V*, where *Q* is the charge stored in the membrane capacitance (*C*) when a voltage (V) is applied. When the applied voltage increases with time, *dQ/dt* = *C* × *dV/dt* = current (i), where *dV/dt* = 100 V/S, and the current captured at the onset of the potential ramp for only the lipid bilayer (no xenon) was 2.4*10^–6^ Amps, as shown in Fig. 1. *Cm* was then calculated and had a value of 24 nF (11.4 mF /m^2^ for an area 2.1 × 10^−6^m^2^. It should be mentioned here that the initial step in the bilayer formation process confirms the presence of a lipid bilayer. If a bilayer is not formed, the capacitance at the start of the trace will be in the range of hundreds of nF, indicating the capacitance of a bare electrode without a bilayer (see Appendix B) (Alobeedallah et al. 2020).

For tBLM with incorporated xenon, the *Cm* was found to be 20 nF (10 mF /m^2^ for an area 2.1 × 10^−6^m^2^) (both 10 and 30% xenon had approximately similar *Cm* values). The decrease in the tBLM capacitance upon the addition of xenon is presumably due to an increase in the membrane thickness. The capacitance and conductance data suggest that xenon molecules affect the internal physical structure of tBLM and consequently also alter the electrical conduction properties of bilayers.

**Fig. 3:**
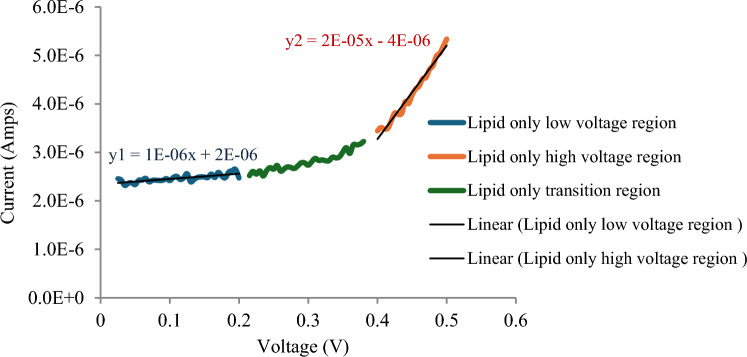
V-I curves of T10 (10% tethers) Am199 bilayers divided into three regions on the basis of linearity: the low-voltage region, the transition region and the high-voltage region

**Fig. 4:**
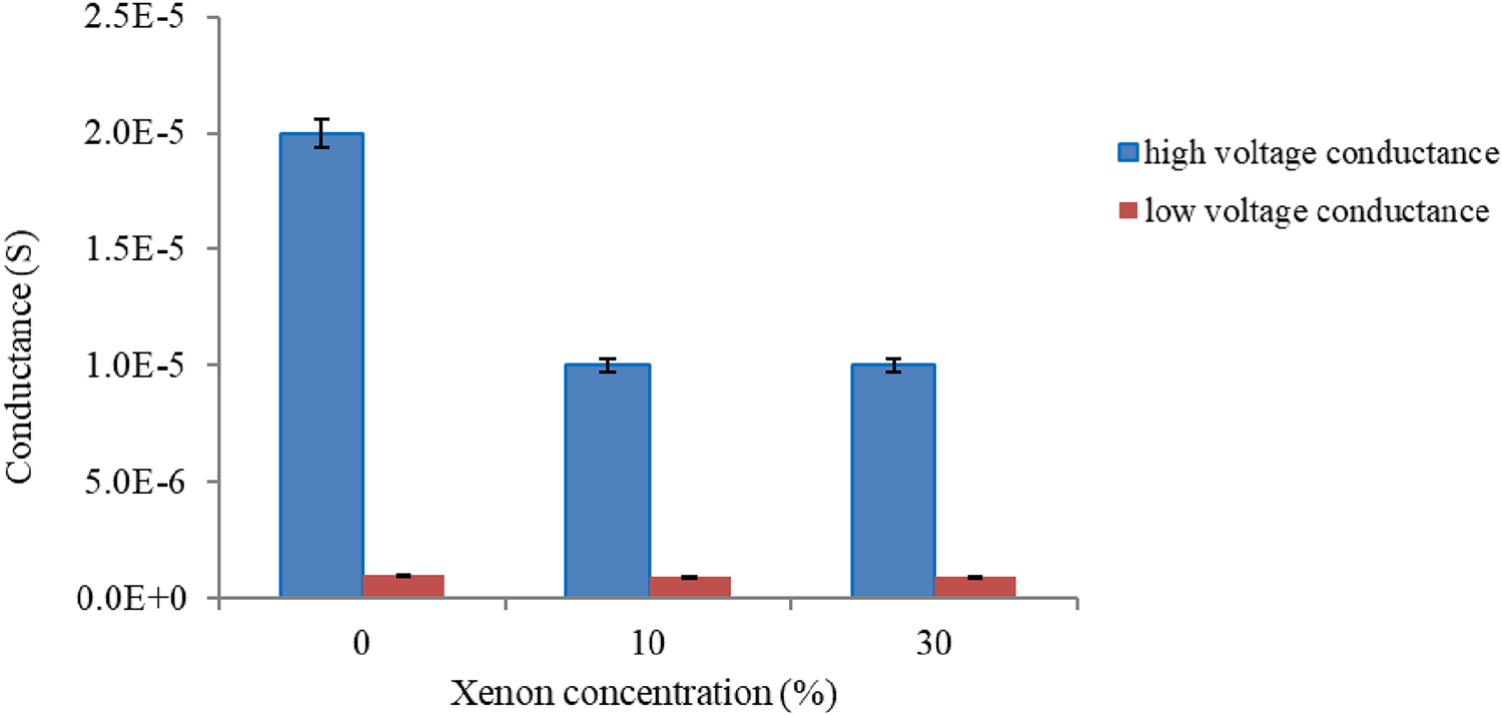
Effects of xenon on the low- and high-voltage conductance of T10 (10% tethers) Am199 bilayers

To study the effect of xenon on pore formation, the V‒I curve was divided into three regions on the basis of its linearity: the low-voltage region, the transition region and the high-voltage region, as shown in Fig. 3. Figure 3 shows that both the low-voltage region and the high-voltage region had approximately linear V-I characteristics. Graphs of the conductance of tBLM with and without xenon at low and high voltages are presented in Fig. 4.

Figure 4 indicates that the membrane conductance at low voltage is not substantially affected by xenon. At high voltage, the membrane conductance is decreased by xenon, but at higher concentrations, it does not further decrease with increasing xenon.

The V–I characteristic curves revealed that the general anaesthetic xenon had the opposite effect to the local anaesthetic Benzyl Alcohol (BZA) on the membrane conductivity of tBLM (Alobeedallah et al. 2016). BZA was found to increases membrane conductivity while xenon tends to decrease the membrane conductivity. This is consistent with the action of many general anaesthetics, which tend to reduce membrane conductivity.

As mentioned previously, xenon has a strong affinity for hydrophobic environments. Therefore, it preferentially localises in the hydrophobic core of the bilayer below the lipid head groups (Pope et al. 1986). The localisation of xenon in the hydrophobic core of the bilayer has substantial effects on the lipid hydrophobic acyl chains. Booker and Sum (2013) and Stimson et al. (2005) reported that xenon molecules create a more constrained and ordered environment for lipid hydrophobic acyl chains, increasing the ordering parameter of the acyl chains. This change is normally associated with increased lipid packing and a decreased area per lipid molecule. Thus, xenon affects lipid bilayers by increasing the bilayer thickness and decreasing the area per lipid. This increase in thickness can be entirely attributed to an increase in the gap between the terminal carbons.

The condition for equilibrium between the entropy and energy for the lipid molecules forming lipid bilayers and the lipid monomers can also be used to explain the effect of xenon on lipid bilayers. The lipids packed in the bilayer are in chemical equilibrium with lipid monomers. The chemical potential for the molecules in these two states must be the same. The equilibrium condition can be simplified to:

1 $$\mu _{1,0} + kT {\text{ln }}X_{1} = \mu m_{,0} + \gamma m a,$$

where *μ*_1*,*0_ and *μm,*_0_ are the standard chemical potentials for the monomer and bilayer, respectively. *X*_*1*_ is the mole fraction of the monomer, *a* is the surface area per molecule, and *γ*_*m*_ is the interfacial free energy per unit area. The interfacial free energy of the membrane in contact with aqueous media, *γ*_*m,*_ is a very important parameter that affects the stability and electrical properties of the membrane (Coster 2003). The value of *γ*_*m*_ of such a bilayer is given by Eq. (2):

2 $$\gamma m = \frac{{\mu _{1,0} - \mu m_{,0} + kT {\text{ln }}X_{1} }}{a}$$

As discussed earlier, the absorption of xenon molecules into the lipid bilayer is expected to decrease the area per lipid molecule. Therefore, a decrease in the area term on the right-hand side of Eq. (1) will occur. To maintain the equilibrium in the chemical potential between the two states of lipids, the interfacial free energy of the membrane surface *γ*_*m*_ is expected to increase. Thus, xenon can affect the electrical properties of tBLM by increasing the interfacial free energy of the membrane surface. By increasing the interfacial free energy, xenon could potentially alter the formation of pores in the lipid bilayer structure and hence electrical conductivity of the membrane, which is dominated by the presence of and size, of such pores. In addition, according to Eq. (1), the decrease in the surface area per molecule is also expected to cause an increase in the standard chemical potential for the bilayer *μm,*_0_ to maintain equilibrium between the two states.

### Effect of Xenon on Tethered Lipid Membranes with Different Tethering Densities

Previous work on the effect of xenon on tBLM used T10 (10% tethers) tethered membranes. The effects of xenon on high-tether-density T100 membranes (100% tethers) and on low-tether-density T1 membranes (1% tethers) are presented.

**Fig. 5:**
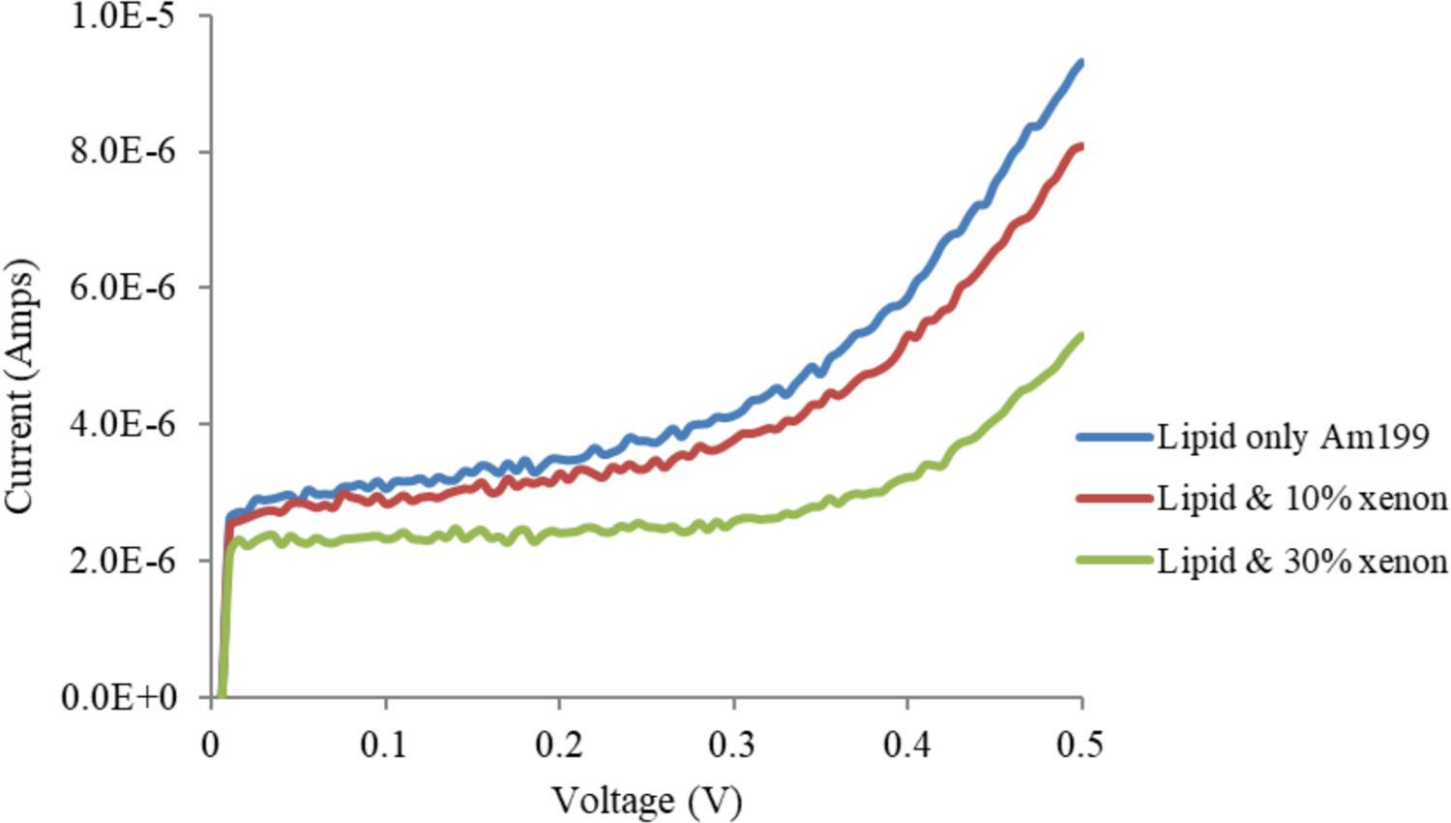
Effects of xenon on T1 (1% tethers) Am199 bilayers

**Fig. 6:**
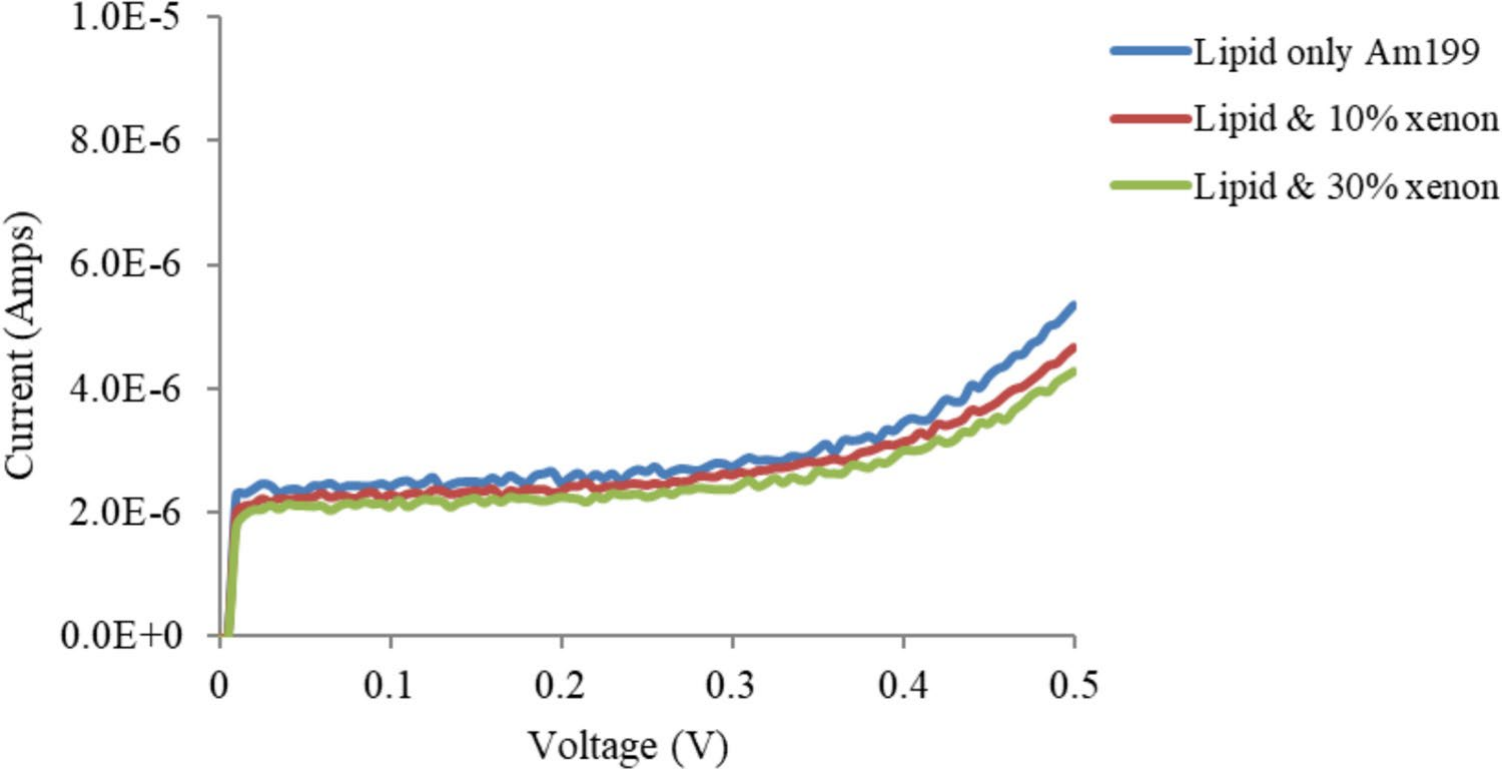
Effects of xenon on T10 (10% tethers) Am199 bilayers. This is a representation of Fig. 2 at different scales to aid in comparison with the 1 and 100% tether data

**Fig. 7:**
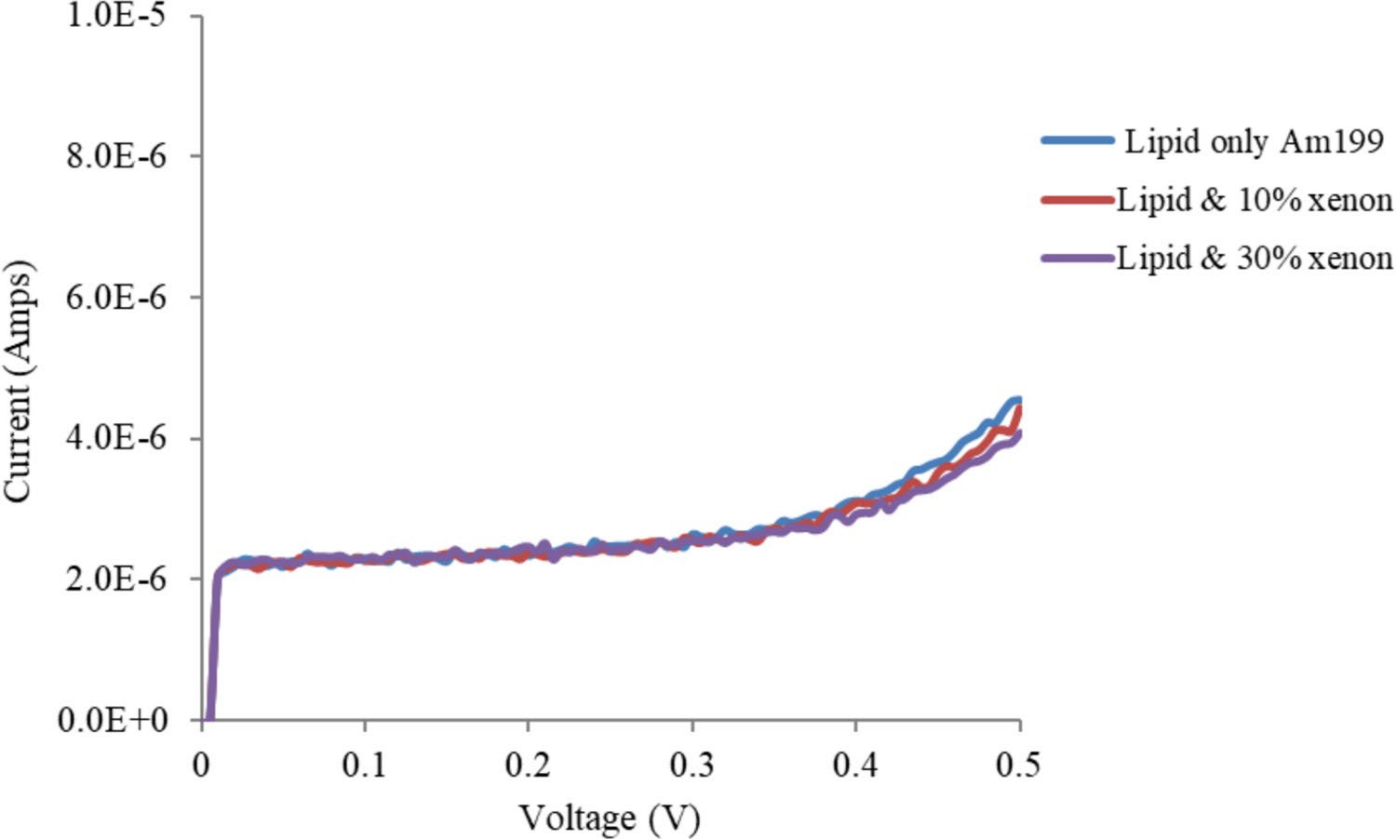
Effects of xenon on T100 (100% tethers) Am199 bilayers

Figures 5, 6 and 7 show the effects of xenon on T1 (1% tethers), T10 (10% tethers) and T100 (100% tethers) tethered membranes, respectively. Xenon reduced the conductivity of all the membranes. However, as the tethering density decreased, xenon had a greater effect on tBLM. This effect was most noticeable on the T1 tethered membranes. Xenon had a lesser effect on the T10 membrane than on the T1 membrane and had the lowest effect on the T100 membrane. Xenon is not expected to induce swelling of the membrane when the lipid bilayers are fully tethered, (T100) as the tethers restrict membrane expansion. As the tethering density of the membrane increases, fewer and smaller pores exist in the tBLM; hence, the T100 bilayers have almost no pores or at least very small pores that cannot grow when voltages are applied. At higher tethering densities, the lateral movement of lipids and gramicidin molecules is inhibited. This interferes with the dimerisation of gramicidin to form conductive channels. Xenon also affects the pores in the membrane, and a greater tethering density further restricts the lateral diffusion properties of gramicidin and the dimerisation process. Thus, xenon is expected to have a reduced effect on the conductance of these membranes.

To obtain further details on pore formation and sizes in tBLM with incorporated xenon, conductance‒temperature dependence measurements were performed, as discussed below.

### Conductance–Temperature Dependence Measurements

**Fig. 8:**
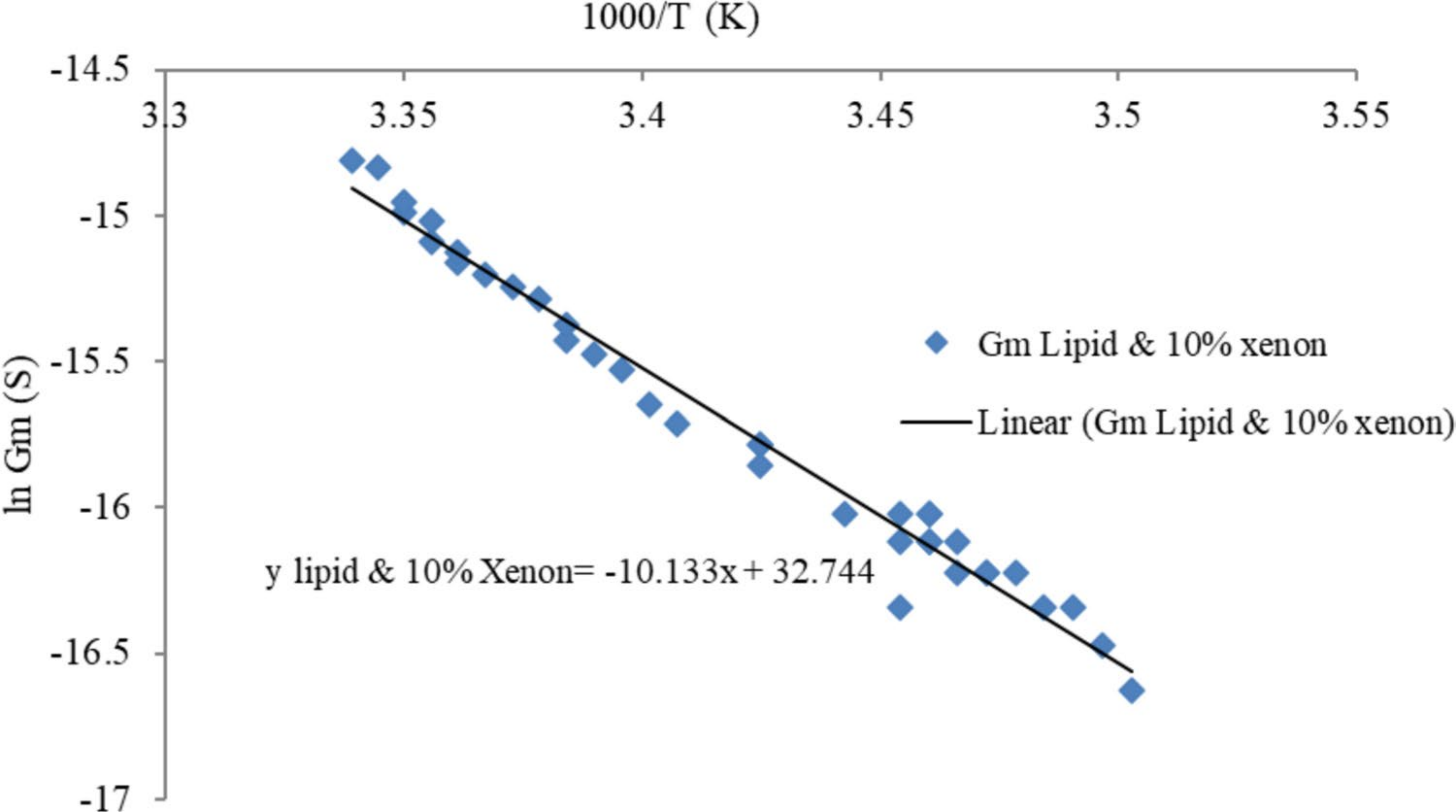
Conductance–temperature dependence curves of tethered lipid bilayers with xenon

The conductance–temperature dependence curve of tethered lipid membranes with incorporated xenon is shown in Fig. 8. These data were used to calculate the activation energy for electrical conduction through lipid bilayers with incorporated xenon, which was then used to calculate the properties of the membrane pores. The activation energy and the Born energy were calculated via Parsegian’s model of the translocation of ions through existing transmembrane pores. The methods used to calculate the born energy and the membrane pore properties were explained in detail in our previous work (Alobeedallah et al. 2022a, b). A brief summary of the method is also included in Appendix A.

### The Conductance–Temperature Dependence Measurements of Tethered Lipid Membranes with Incorporated Xenon at Low Voltage

**Fig. 9:**
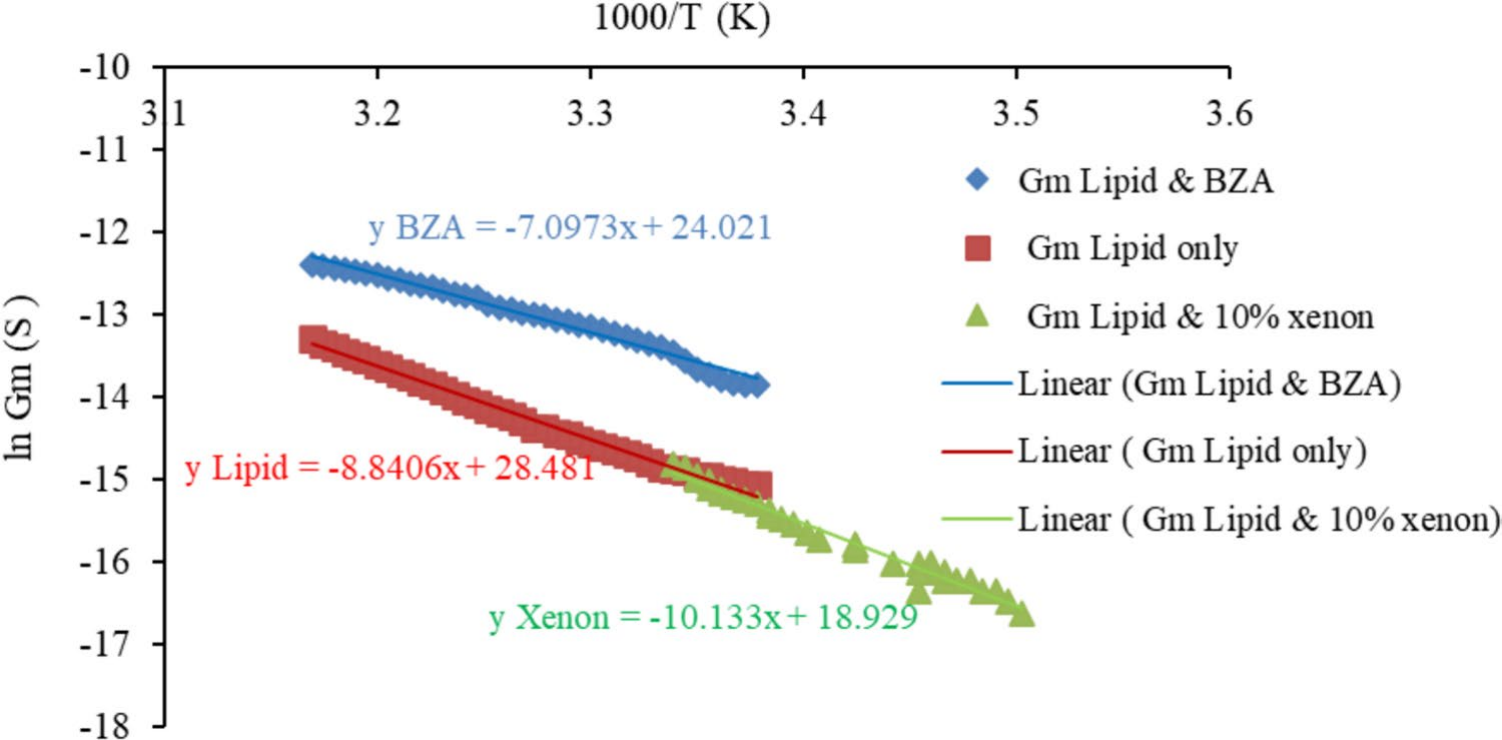
Conductance–temperature dependence curves of tethered lipid-only bilayers at low voltages, tethered lipid bilayers with xenon and tethered lipid bilayers with BZA

Figure 9 presents the Conductance–temperature dependence curves of tethered lipid-only bilayers at low voltages, tethered lipid bilayers with xenon and tethered lipid bilayers with BZA.

The activation energy for charge translocation can be calculated via the Arrhenius equation:

3 $$k = A e^{{\frac{ - Ea}{{RT}}}},$$

where *k* is the Arrhenius rate constant, *Ea* is the activation energy, *R* is the gas constant, and *T* is the temperature in Kelvin. A plot of ln* k* versus *1/T* then yields $\frac{Ea}{RT}$ from the slope.

The conductance–temperature dependence curve of tBLM with incorporated xenon had a slope of approximately 10, as shown in Fig. 8. The total activation energy (Ea) for electrodiffusion through a tBLM with 10% xenon was then calculated via Eq. (3) and found to be ~ 84 kJ/mol. From this, the Born energy was calculated and found to be ~ 66 kJ/mol (see Appendix A). The Born energy of tBLM with incorporated xenon is greater than that of lipid-only bilayers, which was previously calculated as ~ 55 kJ/mol (Alobeedallah et al. 2022a, b). The average pore size in the tBLM structure was then calculated and found to be ~ 3*10^–11^ m (see Appendix A). This is almost tenfold smaller than the pores in tBLM alone and in tBLM with 50 mM BZA. Therefore, the increase in the activation energy for electrical conduction through lipid bilayers with incorporated xenon implies a small pore size. Moreover, the localisation of xenon molecules within the hydrophobic core of the lipid bilayer increases the hydrophobic nonconductive properties within the bilayers due to the inert hydrophobic properties of xenon molecules, which could lead to an increase in the activation energy.

For a 5 nm thick lipid bilayer with incorporated xenon, the partition coefficient ${\gamma }_{\text{partition}}$ was calculated and found to be ~ 2*10^–12^ (see Appendix A). Next, the conductivity of the pore ${G}_{p}$ was calculated and found to be ~ 9*10^–19^ S/m^2^ (surface area 2.1*10^–6^ m^2^), which is much lower than the conductivity of lipid bilayers with 50 mM BZA at 2*10^–16^ S/m^2^ (surface area 2.1*10^–6^ m^2^) (see Appendix A).

${G}_{m}$ for tBLM with incorporated xenon was obtained from the slope of the V–I curve at low voltage shown in Fig. 3. ${G}_{m}$ was found to be 9*10^–7^ S (0.4 S/m^2^). The number of pores that exist in the lipid bilayer with incorporated xenon was then calculated and found to be 2*10^23^ pores/m^2^ (surface area 2.1*10^–6^ m^2^).

Table 1 presents a comparison between the activation energy data of lipids only, lipids and 50 mM BZA and the results of Xenon’s work.

**Table 1:** The activation energy and pore properties of tethered membranes, tethered membranes with 50 mM BZA and tethered lipid membranes with 10% xenon

Tethered lipid membrane	Activation energy (j/mol) ± 4*10^3^	Specific conductivity of the pores (s/m^2^) ± 1*10^–16^	Number of pores (pores/m^2)^ ± 4*10^16^	Average pore radius (m) ± 2*10^–11^
Only lipid (Alobeedallah et al. 2022a, b)	∼73 *10^3^	∼5*10^–17^	∼1*10^16^	~ 2*10^–10^
Lipid & 50 mM BZA (lipid & local anaesthetic) (Alobeedallah et al. 2023)	~ 59 *10^3^	~ 2*10^–16^	~ 1*10^16^	~ 3* 10^–10^
Lipid &10% Xenon (lipid & general anaesthtic)	∼84 *10^3^	~ 9*10^–19^	~ 2*10^23^	~ 3*10^–11^

### The Conductance–Temperature Dependence Measurements of Tethered Lipid Membranes with Incorporated Xenon at High Voltage

The V‒I curves of tBLM with xenon were measured at various temperatures via the same cooling protocol discussed earlier. This experiment was conducted to calculate the electrical properties of tBLM with incorporated xenon in the high-voltage region of the V‒I curve (Fig. 9).

**Fig. 10:**
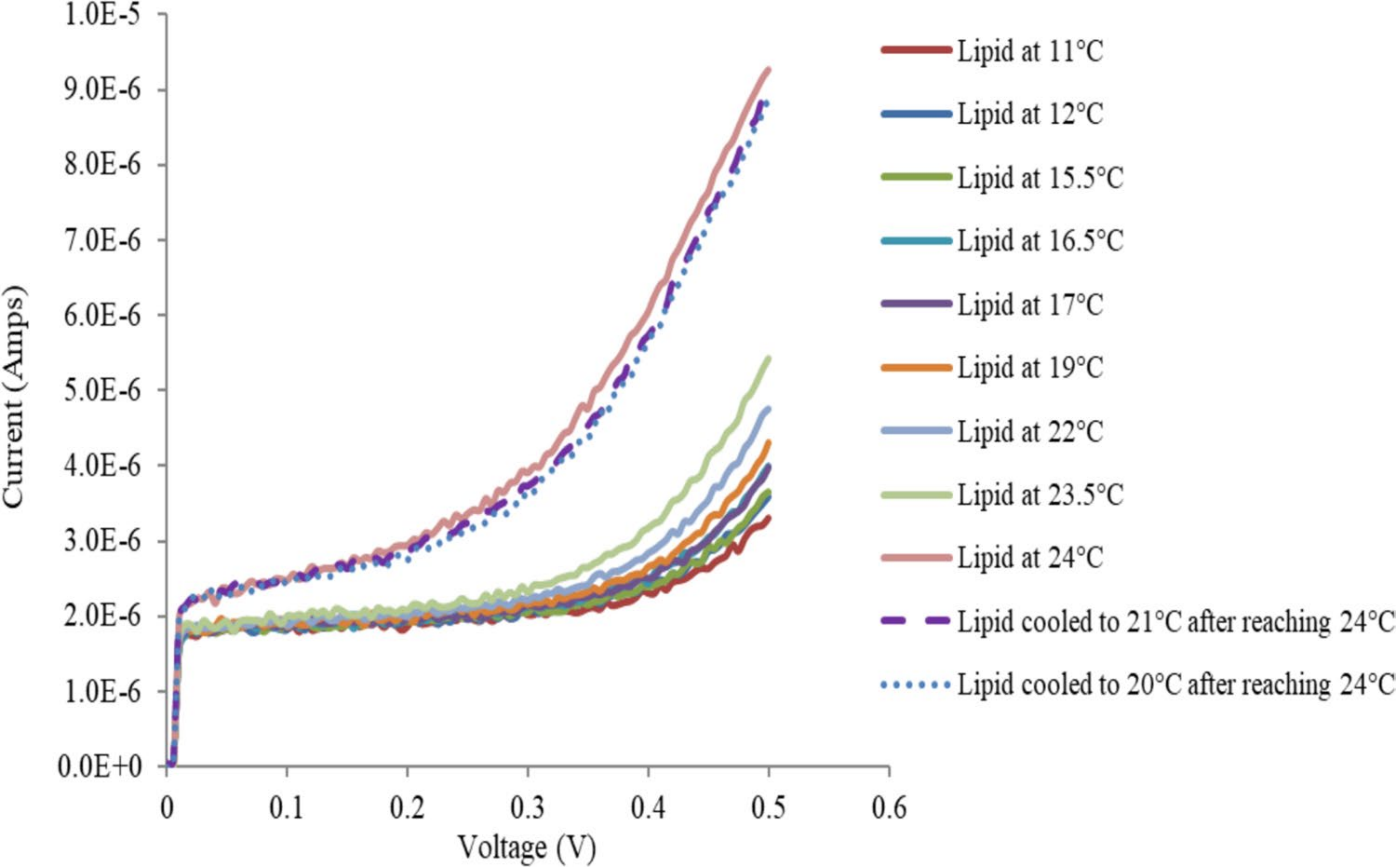
Effects of temperature on the V‒I curves of T10 (10% tethers) AM199 bilayers with Xenon

Figure 10 shows the V-I curves of tBLM with incorporated xenon at different temperatures. The final regions (at high voltage) of the V‒I curves of tBLM with xenon at different temperatures were all parallel. This indicates that the activation energy at high applied voltages is approximately constant. Thus, neither the activation energy nor the pore properties could be calculated via V-I curves at various temperatures. This outcome is similar to earlier conclusions on the activation energy of tBLM (with incorporated BZA, cholesterol or lipids alone) in the high-voltage region (Alobeedallah et al. 2023). These results shed light on the important role that the potential difference across the biological membrane can play in determining the permeability of the membranes, consequently controlling many biological mechanisms.

Figure 10 also shows a sudden increase in the current through tethered membranes with incorporated xenon at approximately 24 °C during the warming of the membrane. The membrane/xenon system was subsequently cooled to temperatures less than 24 °C (21 and 20 °C) in an attempt to recover the original V‒I curves of the tBLM with xenon measured at lower temperatures; however, the original V‒I curves from the tBLM before the sudden increase in the current were not recovered. This observation may be due to the total loss of xenon from the membrane at this relatively high temperature. This experimental observation highlights the fact that the solubility of xenon in lipid membranes is very sensitive to temperature. Therefore, the conductance–temperature plots are approximations only because at different temperatures, the membranes contain different amounts of xenon.

## Conclusion

Tethered lipid membranes with xenon incorporated within them were constructed. V-I measurements were performed to study the effects of xenon on the electrical properties of tBLM (Am199). The results showed that incorporating xenon into Am199 *decreased* the membrane conductance. The effects of xenon on lipid bilayers with different tethering densities were also investigated. Xenon affected all these membranes by reducing their conductivity. However, xenon had the strongest and most detectable effect on the T1 (1% tethers)-tethered membranes, whereas it had the least effect on the T100 (100% tethers)-tethered lipid bilayers. As the tethering density of the membranes increases, less space will be available between the tethers. Therefore, with high tethering density, the formation of pores in the lipid bilayer is limited.

The results of the conductance–temperature dependency of tBLM with xenon were presented at low and high voltages. Xenon was found to increase the activation energy of electrical conduction through bilayers, as well as the Born energy. Furthermore, xenon decreased the average pore size in tBLM at low applied voltages.

### Conjecture on the Effect of Xenon on Anaesthesia

Anaesthesia is related to the generation of action potentials in nerves. In terms of the Hodgkin–Huxley–Katz description of nerve excitation, the action potential requires the opening (and then closure) of sodium channels. The sodium channels are voltage-gated protein ion channels composed of 6 transmembrane subunits. Coster (2003) reported that the lateral aggregation of the subunits in the lipid bilayer is promoted by a partial mismatch between the hydrophobic portions of the subunits and that of the lipid bilayer; this hydrophobic mismatch leads to lateral aggregation. We have shown in this study that xenon causes an increase in the Born energy for ion translocation through pores that results from changes in the hydrophobicity of the membrane, which affects the hydrophobic lateral aggregation of ion channel subunits.

## Figures and Tables

**Fig. 11:**
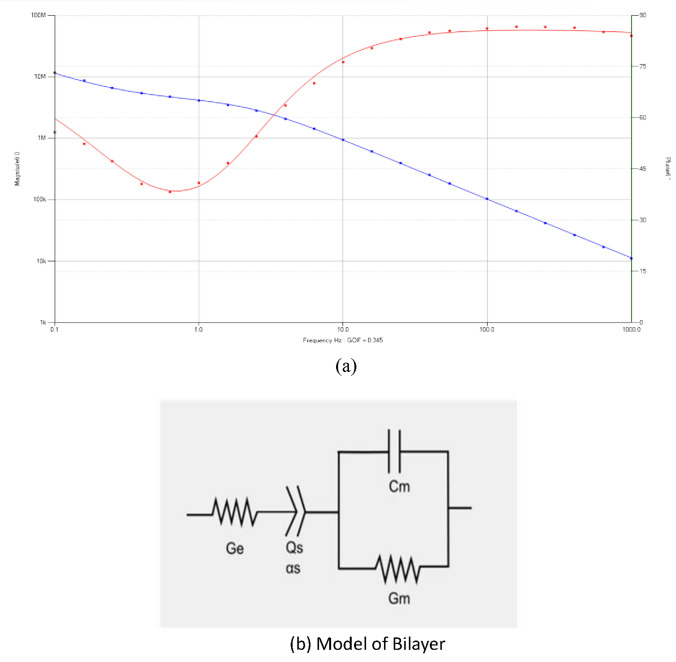
**a** The phase and magnitude Bode Blots for tethered lipid membranes. **b** The equivalent circuit model representing a lipid membrane system

## Data Availability

No datasets were generated or analysed during the current study.

## Appendix A

1. The total activation energy for ionic conduction through a pore is given by: ${E}_{a = }{E}_{D} + {E}_{B},$ where ${E}_{a}$ is the total activation energy, *E*_*B*_ is the Born energy and ${E}_{D}$ is the energy required for diffusion through the aqueous medium filling the pore, which is ∼18 kJ/mol.
2. Using the experimentally determined activation energy for conduction *E*_*a*_, the Born energy ${E}_{B}$ can be calculated.
3. The Born energy for inserting an ion into a pore is given by ${E}_{B= }\frac{{Z}^{2} {q}^{2 }\alpha }{4 \pi {\varepsilon }_{0 }{\varepsilon }_{h}r}$, where α =  ~ 0.2 for a long cylindrical pore. From this, the average pore radius $\text{r}$ was calculated.
4. The average pore conductivity can then be calculated via the following equation:
  $$\text{Gp}= \sigma \frac{A}{L}= \sigma \frac{\pi {r}^{2}}{d},$$
  where Gp is the conductivity of the pore, *d* is the thickness of the bilayer and $\sigma$ is the specific conductivity inside the pore.
5. The specific conductivity inside the pore ($\sigma$) is calculated via the following equation:
  $$\sigma = \sigma_{{\text{Pbs *}}} \gamma _{{{\text{partition}}}},$$
  where ${\upsigma }_{\text{Pbs}}$ represents the specific conductivity of the PBS solution, which was 17 × 10^–3^ S/cm, and where ${\upgamma }_{\text{partition}}$ represents the partition coefficient. Therefore:
  $$\text{Gp}= \sigma \frac{A}{L}=\left({\sigma }_{\text{Pbs}*}{\gamma }_{\text{partition}}\right)\times \frac{\pi {r}^{2}}{d}$$
6. To calculate the partition coefficient ${\gamma }_{\text{partition}}$, the following equation was used:
  $${\gamma }_{\text{partition}}= {e}^{\frac{-{E}_{B}}{KT}}$$
7. The total membrane conductance ${G}_{\text{m}}$ is the total number of pores $n\left(r\right)$ multiplied by the specific conductance of each pore ${G}_{\text{p}}:$
  $${G}_{m }= n\left(r\right)\times {G}_{p}$$

## Appendix B

By fitting the Bode plots of the impedance data to an equivalent circuit model—representing a bilayer in series with a capacitive gold electrode—the membrane capacitance was determined to be approximately 1 µF/cm^2^ (10 mF /m^2^), a widely accepted value for a bilayer. This value was maintained throughout all experiments with minor variations experienced on the addition of xenon. If there was no membrane the capacitance would be × 10 larger and the Bode plot would not have a minimum. Figure 11 presents the phase and magnitude Bode plots for tethered lipid membranes, along with the corresponding equivalent circuit model used to represent the electrical properties of the lipid membrane system (Fig. 11).
